# Model of qualification for physical therapy program: experiences from the Mental Health Support Centre in Tarnowskie Góry, Poland.

**DOI:** 10.1192/j.eurpsy.2023.2186

**Published:** 2023-07-19

**Authors:** A. R. Szczegielniak, J. Smolarczyk, R. Pudlo

**Affiliations:** Department of Psychoprophylaxis, Medical University of Silesia in Katowice, Katowice, Poland

## Abstract

**Introduction:**

In terms of physical and mental health benefits, psychiatric rehabilitation requires diverse therapeutic activities offered by an interdisciplinary team. In order for patients to be able to fully participate in social life, it is necessary to strengthen health-promoting activities, control the symptoms and side effects of pharmacological treatment, effectively manage resources and counteract interpersonal and environmental barriers caused by disability. Physical activity and exercise programs answer both psychosocial and biological aspects of health leading to higher self-efficacy, lower self-perceived stigma, longer lifespan and overall better quality of life

**Objectives:**

The aim of this study is to present qualification process of adult patients for physical therapy program at the Mental Health Support Centre’s (Centrum Wsparcia Zdrowia Psychicznego, CWZP) daily rehabilitation unit in Tarnowskie Góry.

**Methods:**

Adult patients with diverse diagnoses (schizophrenia, affective disorders, anxiety disorders or organic mental disorders) and varied degree of functioning who met the admission criteria were accepted to the ward for a period of 12 weeks. During the stay, a wide range of therapeutic activities was offered, including individual psychological support, group work, art therapy, relaxation sessions, culinary/ dietary workshops, individual training and general fitness group exercises, as well as cognitive training (also via computer-based programs). Due to the COVID-19 epidemic, some of the activities have been limited.

**Results:**

Physical exercises, just as any treatment, should be offered in appropriate doses. Patients with mental disorders, especially severe mental illnesses, experience many barriers in engaging in physical activity and are under a greater risk of sedentary lifestyle. Thus, for the qualification, exercise tolerance assessment was performed in the form of 6-min Walk Test (6MWT) with Borg scale for subjective fatigue. Through aerobic capacity and endurance assessment patients’ respiratory system, cardiovascular system, and neuromuscular system functions can be evaluated. Functional fitness was assessed through 3 elements of Senior Fitness Test (SFT) (Back Scratch test, Up and go test, Chair Sit-and-Reach test) along with Romberg Test to evaluate the static and dynamic balance as well as flexibility of the upper and lower body.

**Conclusions:**

Properly planned and carried out qualification for the physical therapy program allows to adjust the activities to the needs of the patient. Additionally, it can be a tool to evaluate the achieved results at the end of the rehabilitation process.

**Disclosure of Interest:**

None Declared

